# Pleasure and suffering of nursing in COVID-19 hospital units: between disenchantment and formation of meanings

**DOI:** 10.1590/0034-7167-2022-0356

**Published:** 2023-05-08

**Authors:** Alexa Pupiara Flores Coelho Centenaro, Andressa de Andrade, Gianfábio Pimentel Franco, Rosângela Marion da Silva, Leticia Silveira Cardoso, Lílian Moura de Lima Spagnolo, Clarice Alves Bonow, Marta Cocco da Costa

**Affiliations:** I Universidade Federal de Santa Maria. Palmeira das Missões, Rio Grande do Sul, Brazil; II Universidade Federal de Santa Maria. Santa Maria, Rio Grande do Sul, Brazil; III Universidade Federal do Pampa. Uruguaiana, Rio Grande do Sul, Brazil; IV Universidade Federal de Pelotas. Pelotas, Rio Grande do Sul, Brazil

**Keywords:** Nursing, Occupational Health, COVID-19, Nurse Practitioners, Hospital Units, Enfermería, Salud Laboral, COVID-19, Enfermeras Practicantes, Unidades Hospitalarias, Enfermagem, Saúde do Trabalhador, COVID-19, Profissionais de Enfermagem, Unidades Hospitalares

## Abstract

**Objective:**

to analyze the experiences of pleasure and suffering of nursing workers in COVID-19 hospital units.

**Methods:**

a multicenter, qualitative study, developed with 35 nursing workers from COVID-19 units in seven hospitals in southern Brazil. Data were produced through semi-structured interviews, submitted to thematic content analysis with the help of NVivo.

**Results:**

experiences of pleasure were linked to gratification, identification with work content, positive results in care, recognition, integration with the team and personal overcoming. Suffering was revealed in daily life of deaths and losses, feelings of helplessness, team conflicts, institutional demands, professional devaluation. Workers reported disenchantment, but also strengthening the meaning of their work, highlighting frontline impacts on their mental health.

**Final considerations:**

in the dynamics between pleasure and suffering in nursing work in COVID-19 hospital units, elements point to the risk of psychological illness.

## RESUMO

**Objetivo:**

analisar as vivências de prazer e sofrimento de trabalhadores de enfermagem em unidades hospitalares COVID-19.

**Métodos:**

estudo multicêntrico, qualitativo, desenvolvido com 35 trabalhadores de enfermagem de unidades COVID-19 de sete hospitais do Sul do Brasil. Os dados foram produzidos por meio de entrevistas semiestruturadas, submetidos à análise temática de conteúdo com auxílio do *software* NVivo.

**Resultados:**

vivências de prazer estavam vinculadas à gratificação, identificação com o conteúdo do trabalho, resultados positivos na assistência, reconhecimento, integração com a equipe e superações pessoais. O sofrimento se relevou no cotidiano de mortes e perdas, sentimento de impotência, conflitos na equipe, cobranças institucionais, desvalorização profissional. Os trabalhadores relataram desencantos, mas também fortalecimento dos sentidos do seu trabalho, ressaltando impactos da linha de frente em sua saúde mental.

**Considerações finais:**

na dinâmica entre prazer e sofrimento no trabalho de enfermagem em unidades hospitalares COVID-19, elementos sinalizam para o risco de adoecimento psíquico.

## RESUMEN

**Objetivo:**

analizar las experiencias de placer y sufrimiento de trabajadores de enfermería en unidades hospitalarias COVID-19.

**Métodos:**

estudio multicéntrico, cualitativo, desarrollado con 35 trabajadores de enfermería de unidades COVID-19 de siete hospitales del sur de Brasil. Los datos fueron producidos a través de entrevistas semiestructuradas, sometidas al análisis de contenido temático con el auxilio del software NVivo.

**Resultados:**

las experiencias de placer se vincularon a la gratificación, identificación con el contenido del trabajo, resultados positivos en el cuidado, reconocimiento, integración con el equipo y superación personal. El sufrimiento se reveló en el cotidiano de muertes y pérdidas, sentimientos de impotencia, conflictos de equipo, exigencias institucionales, desvalorización profesional. Los trabajadores informaron desencanto, pero también fortalecieron el significado de su trabajo, destacando los impactos de primera línea en su salud mental.

**Consideraciones finales:**

en la dinámica entre placer y sufrimiento en el trabajo de enfermería en unidades hospitalarias COVID-19, elementos apuntan para el riesgo de enfermedad psíquica.

## INTRODUCTION

Nursing is the most numerous workforce in health in Brazil and in the world. It plays a fundamental role in establishing direct care^(1)^, offering promotion, protection and treatment of health problems for the population. However, there are stressful elements in their daily lives, such as the complexity of interpersonal relationships, in addition to the overload of activities and technical responsibility, which can weaken their mental health. These elements were strengthened with the advent of coronavirus disease 2019 (COVID-19)^(2)^, a disease caused by the Severe Scute Respiratory Syndrome Coronavirus 2 (SARS-CoV-2).

Acting on the front line of care for patients affected by COVID-19 exposes nursing workers to risk situations for infection by SARS-CoV-2^(3)^. Furthermore overwork, isolation, lack of contact with one’s own family and physical and emotional exhaustion arising from daily life with critically ill patients pose risks to their mental health^(4)^.

For this reason, it is important to know the relationships that are established between frontline work and its subjectivity. One way to do this is to identify the experiences of pleasure and suffering at work, concepts developed by Psychodynamics of Work (PDW), French current that studies the ways in which the phenomena that occur in work relationships interfere with workers’ subjectivity and mental health^(5)^.

Experiences of pleasure are the result of positive dimensions that structure the work context. They are manifested through personal gratification, achievement, recognition, freedom and appreciation. They strengthen mental health, as they make it possible to structure workers’ identity and create synergy between individuals’ subjectivity and work’s concrete reality. The experiences of suffering, on the other hand, are the result of mismatches between the desires, aspirations and expectations of individuals and real work, i.e., daily life marked by elements that are beyond what is prescribed or expected. In addition, suffering manifests itself when professionals do not find spaces of freedom within the organization of work that allow them to express their individuality and transform the object according to their wishes^(5-7)^.

According to the PDW, experiences of pleasure operate as mediators of mental health and the meaning of work. However, the memory of suffering constitutes a force capable of mobilizing subjects towards the transformation of work organization. Thus, pleasure and suffering are coexisting everyday experiences. Health or psychological illness depend on the dynamics between these feelings and, above all, on individuals’ ability to transform their work organization and to reframe experiences of suffering into pleasure^(5-6)^.

The health workforce is one of the most important resources for effectively coping with COVID-19^(8)^. However, being on the front line increases the risk of psychological distress and mental illness, especially among nursing workers. Thus, recognizing the relationships between work, subjectivity and health will enable the implementation of actions that mitigate the negative impacts of the pandemic on the category^(9)^. In addition, there are no studies that analyze the experiences of pleasure and suffering of nursing workers specifically in COVID-19 hospital units, which justifies the relevance of this study.

## OBJECTIVE

To analyze the experiences of pleasure and suffering of nursing workers in COVID-19 hospital units.

## METHODS

### Ethical aspects

The ethical aspects of research with human beings recommended by the Brazilian National Health Council were complied with, in accordance with Resolutions 466/2012 and 510/2016. The project was approved by the local Research Ethics Committee.

### Study design and setting

This is a multicenter, qualitative and descriptive study, conducted in accordance with the COnsolidated criteria for REporting Qualitative research (COREQ) guidelines. It was carried out in seven medium and large hospital institutions located in different mesoregions of Rio Grande do Sul, Brazil (Northwest, Central-West, Central-Eastern, Metropolitan, Southeast and Southwest). Five were philanthropic and two were public hospitals linked to federal educational institutions. The scenarios were sectors that received patients with suspected or confirmed COVID-19: a respiratory triage unit; five emergency sectors; four clinical inpatient units; and four Intensive Care Units (ICUs).

The choice for different scenarios with regard to geographic location, size and forms of administration/management aimed to capture the different possible experiences in the state of Rio Grande do Sul. The diversity in the composition of the settings enriched the findings of this study, as it enabled the identification of counterpoints between different views. However, it should be noted that the choice of COVID-19 units established an approximation of these scenarios with reference to the nature of work, which made it possible to homogenize the findings.

### Participants and eligibility criteria

Nursing workers (nurse, technician or assistant) working in a COVID-19 unit, performing care activities on the front line of patient care were included. Workers on vacation or any type of functional leave were excluded.

Institutions provided lists with names and email and/or telephone contacts of professionals who met the eligibility criteria (470 in total). It was decided to draw five workers from each institution, so that there would be equal representation in the study composition. This was the initial contingent of participants to trigger data production.

When carrying out the draws, twelve workers did not respond to the researchers, even when contacted three times. In addition, there were five refusals, whose main reasons mentioned were excessive work and research. In these cases (no response or refusals), new draws were carried out until five participants per institution were reached.

Once this representativeness was achieved, data were submitted to pre-analysis of theoretical saturation, through which it was identified that the findings were consistent enough to be considered revelations of the group, allowing inferences about them^(10)^. Therefore, the composition of a group of participants with 35 respondents (i.e., five from each institution) was completed.

### Data production

Data were produced between September 2020 and July 2021 through individual semi-structured interviews. They were carried out by a group of professors linked to public institutions, with a doctoral degree, with experience in collecting qualitative data, in addition to two previously trained master’s students from a graduate nursing program. The contact with the drawn participants was mediated by the nursing heads.

In five institutions, the interviews took place on-site at the workplace. Safe, private spaces were used and biosecurity measures were maintained. After reading and signing the Informed Consent Form (ICF), data were collected to characterize the participants (gender, age, color/race, professional category, unit/sector where they were working). Next, the interview was conducted based on a semi-structured script that focused on perceptions and feelings about working on the front lines of coping with COVID-19.

In two hospitals, interviews were conducted online, at the request of administrators. Participants received the ICF and signed their consent through an electronic form (Google Forms). The meetings took place with the help of Google Meet, a free platform that allows real-time interlocution, with audio and image sharing. Conducting the online interviews was identical to that conducted in the face-to-face format. The recording took place through a feature of Google Meet itself.

A semi-structured script was built by the multicenter team especially for this study. The protocol for selecting participants, approaching and conducting the collection was standardized for the seven institutions. The first interview, considered a pilot, showed no need to change the script, so it was included in the study. The interviews were audio-recorded, with participants’ consent, and lasted approximately 22.5 minutes.

It is emphasized that the field stage ended at the end of the 35^th^ interview (obtained from the representativeness of five participants per institution). As the empirical material reached theoretical saturation^(10)^, the corpus was considered sufficient to respond to the objective of this study.

### Data analysis

The thematic content analysis technique was used, which is developed in three stages: pre-analysis; material exploration; treatment of data obtained and interpretation^(11)^.

In pre-analysis, immersion in the empirical content was carried out, through skimming reading, which enabled identifying the content and selecting relevant material for analysis. In material exploration, the material was decomposed and coded with the help of New NVivo Academic, enabling the grouping of analytical categories and subcategories. In treatment of data obtained and interpretation, the researchers focused on the results and performed interpretations and inferences that led to the conclusions^(11)^.

During data analysis, the diversity of experiences of participants was considered, due to the different institutions and units to which they were linked. Therefore, care was taken to pay attention to the plurality of findings, including testimonials in the RU that represented different points of view in relation to each core of meaning.

When presenting the results, respondents are identified by the letter T (which begins the word “worker”) followed by a random cardinal number. There is also the identification of its capacity unit: Emergency or Respiratory Screening Units COVID-19 (COVID-19 Emerg.); COVID-19 Inpatient Units (COVID-19 Inp.); COVID-19 Intensive Care Unit (COVID-19 ICU); and more than one COVID-19 sector (COVID-19 Sectors). The codes also inform if the participant was assigned to a public or philanthropic institution.

## RESULTS

In all, 35 nursing workers participated in the survey. Among them, 80% (n=28) were women, and the mean age was 38 (±8.9) years. With regard to color/race, 77% (n=27) were white and 23% (n=8) black or brown. With regard to professional category, 71.4% (n=25) were nursing technicians and 28.6% (n=10) nurses.

With regard to capacity units, 42.8% (n=15) were in Emergency Units or COVID-19 Respiratory Screening Units, 31.4% (n=11), in COVID-19 Inpatient Units. 19, 22.8% (n=22.8), in a COVID-19 ICU, and 2.8% (n=1), in more than one COVID-19 sector. The results are presented in the following analytical categories.

### Experiences of pleasure in nursing work in COVID-19 hospital units

Participants unveiled a set of feelings that point to experiences of pleasure in the daily work in COVID-19 hospital units. These experiences were common to workers in public and philanthropic institutions, and in all COVID-19 units. They did not fail to recognize the losses they experienced on the front lines of the pandemic, but they considered that this experience also provided personal gratification:

[...] *stressful at times, but rewarding at others. Just as we saw patients who died, we also saw patients who managed to survive. They managed to leave the ICU. Patients who talked exposed even their personal lives. From that point of view, it was cool* [...]. (T29-COVID-19 ICU/philanthropic)

[...] *as much as it is tiring, stressful* [...] *I like it, I’m here by choice. For the salary, of course, and also because I like it.* [...] *I feel important. Seeing those people go through so much and then come out of it well* [...]. (T20-COVID-19 Emerg./public)

Identifying with the nature of work also enhanced the experiences of pleasure, as the care provided to patients with COVID-19 provided participants with an approach to the essence of nursing care:

[...] *it was a return, a reunion with everything I always liked to do. I was feeling frustrated that I was no longer in contact with the patient. I always liked to take care. There is a caregiver, I was born for this* [...]. (T20-COVID-19 Emerg./public)

[...] *I like it, I love working there* [COVID-19 unit]. [...] *I love my job, I love nursing.* [...] *I’ve been in this business for 12 years. I like dealing with the patient, being in the care, being able to help, feeling useful* [...]. (T31-COVID-19 Inp./public)

Workers highlighted that an important part of their experiences of pleasure were related to the positive results of their work, through which they were able to know themselves as an important part of facing the pandemic:

[...] *I think we have a very important and special role in coping. It doesn’t let me give up. We have a very strong potential and great willpower.* [...] *I feel happy to be here* [...]. (T12-COVID-19 Emerg./philanthropic)

[...] *patients are short of breath. I start celebrating his every victory. They arrive here with 15-liter Hudson* [15 l/min], *reduce it to 10, to 8, to 6, to 4. Suddenly they go to the catheter, 5 liters, 4 liters, when they see they have half a liter.* [...] *and when they manage to leave, I celebrate it a lot* [...]. (T33-COVID-19 Inp./public)

There was an emphasis on the recognition obtained by patients, especially in moments when positive results of care were obtained:

[...] *very gratifying to see the patient’s return.* [...] *a guy thanked me, “You certainly made an impact on my life, you helped me, you contributed to my health”. Those words are cool. I feel that gratification, “I was able to help in someone’s life, I managed to make a difference”* [...]. (T14-COVID-19 Emerg./philanthropic)

[...] *it is often rewarding. When we can win this battle. When the day turns and we see their improvement, it is very rewarding. They are grateful.* [...] *they tell us, “You are the angels that protect us”* [...]. (T35-COVID-19 Inp./public)

[...] *when a patient recovers, it touches me a lot. I feel satisfied.* [...] *in mental health, it only made me feel good to work with COVID-19. it’s something impressive.* (T22-COVID-19 Emerg./public)

Workers highlighted the integration and coexistence with co-workers in the sector. Trusting the team made it possible for professionals to feel strengthened in the face of work challenges:

[...] *working in a cohesive team, having competent professionals, empathetic above all.* [...] *I think this is very good for the mental health of those who have to work in a line of work like COVID-19* [unit] [...]. (T33-COVID-19 Inp./public)

[...] *we are very happy. People laugh, joke. Even if we are dealing with a difficult case, even with co-workers positive* [...] *We are facing it, in our own way. That’s why I really like working here* [...]. (T12-COVID-19 Emerg./philanthropic)

[...] *I have a wonderful workgroup.* [...] *people who run after, who want to grow and conquer things. It’s cool, it gives us purpose.* (T20-COVID-19 Emerg./public)

Finally, identifying their process of resilience, overcoming adversity and pride also stood out as an important experience of pleasure:

[...] *I have a small physical disability.* [...] *I stayed away from the hospital; I ended up thinking that I would no longer be able to.* [...] *that was one of my victories. I tell everyone that 2020 was a victorious year, because it was the year I found myself again in my profession.* [...] *I saw that I am capable.* [...] *I evolved a lot in there* [COVID-19 unit]. *My bosses really like my work. This work only brought me happiness* [...]. (T22-COVID-19 Emerg./public)

[...] *the human side blossomed a lot in me. Today, everything I can do for patients, I do.* [...] *I matured as a professional, as a person, as a human being. In addition to the knowledge I sought. I perfected myself in things I didn’t think to pursue before. It was very difficult. There are still many difficulties. But I think it added a lot to our profession and to us as a person* [...]. (T11-COVID-19 Emerg./philanthropic)

*I’m proud to say that I’m from the front line!* (T6-COVID-19 ICU/philanthropic)

The findings show that nursing work, while being challenging, provided intense experiences for professionals, which were able to mobilize feelings of gratification, achievement, overcoming and, therefore, pleasure.

### Experiences of suffering in nursing work in COVID-19 hospital units

In contrast to the data presented so far, the second analytical category highlights the experiences of suffering. Suffering experiences were reported by workers from all types of COVID-19 units, but enunciations from philanthropic institutions predominated. First, references to deaths caused by the disease stood out:

[...] *in the* [surgical] *unit, there are no deaths. There* [COVID-19 unit] *there is. It’s one death a day. Today we lost two patients. It’s impactful, you have to have a very strong psychology to be able to work on it. We have the psychologist for that, she will talk, it’s a blow* [...]. (T6-COVID-19 ICU/philanthropic)

[...] *not all patients who died arrived dying. Many patients who died, the majority, arrived talking, lucid, oriented, communicating. We had a bond with the patient, we talked. We knew he had a son, was married, lived in such a place, and that affected, shook* [...]. (T29-COVID-19 ICU/philanthropic)

After death, those who died from COVID-19 underwent a different protocol that included body preparation by nursing, which caused discomfort for the team. By not allowing family members to say goodbye to their loved one in person, professionals were sensitive to their suffering. Sometimes, they resorted to spiritual comfort, recognizing that they would like to have more power to intervene in the decline of some patients:

[...] *It shocks us a lot when patients die. There is a protocol to prepare the body. It’s quite shocking because we weren’t used to it.* [...] *the body leaves here practically sealed. The only record that the family member has is a photo of dead patients’ face* [...]. (T29-COVID-19 ICU/philanthropic)

[...] *it shakes us a lot to have to prepare the body. Pack it, bag it and take it to the morgue. That last moment that the family should have and cannot have* [...] *to see the person* [...]. (T1-COVID-19 Emerg./philanthropic)

[...] [patient] *arrives with the family talking about the family, the children. After 40 minutes, I lost that person.* [...] *I try to talk about Jesus to people, because I don’t have anything to offer. Asking to remain calm, because I see that anxiety also kills a lot, it makes them end up going to the tube* [...]. (T8-COVID-19 ICU/philanthropic)

Other experiences of suffering were evident beyond the daily care provided to patients. Some nursing professionals revealed the contamination and death of co-workers and their own family members by COVID-19. Professionals felt vulnerable and, at times, guilty and powerless, as if a paradox was established between saving lives on a daily basis and losing the life of a loved one:

[...] *my husband had problems* [...] *morbidly obese, hypertensive and cardiac.* [...] *in reality I blamed myself a lot. So many years working in the hospital, and I didn’t see the symptoms in him. I thought* [it was] *because he got rained on* [...] [sigh] *I thought he got the flu. He had all the symptoms* [crying] *I lost my husband in the pandemic. Two days after my* [positive] *result.* (T7-COVID-19 Inp./philanthropic)

[...] *sometimes it happens with co-workers. They get sick, they go to the ICU. Go to the tube. Some of ours died. It took a toll on my mental health. It reminded me that I am a human being, like any other.* (T12 - COVID-19 Emerg./philanthropic)

Elements linked to the organization and work process also revealed themselves as promoters of experiences of suffering. There was a highlight for the lack of support from some team members in coping with the pandemic:

[...] *the nurses did not cooperate. The vast majority of them. It was very sad. Mainly the day shift staff, they were left there without nurses’ assistance.* [...] *that was a lot of suffering.* [...] *there was a lack of that human part of seeing how I am with the patient,* [if] *he is saturating well. I have a lot of anger. This is something that really hurt me* [...]. (T18-COVID-19 Inp./philanthropic)

[...] *I leave my unit fully dressed. I arrive at the unit where I will receive a patient with COVID-19, my co-worker is not dressed, he is contaminating everything. I just arrived at the ICU to receive a patient with severe COVID-19, the technician took him away without any paraphernalia, then returned to see her patient who was not contaminated with COVID-19. That’s a crime with people* [...]. (T26-COVID-19 Inp./philanthropic)

Participants highlighted the institutional demands and overload, felt too much at a time when they expected management support. Reports indicate that pressure, demands and overload were related to leave and the desire to leave work:

[...] *co-workers all talked about giving up, that it was very bad to work there* [COVID-19 unit]. [...] *I think that many things had to be highlighted, but not as charged as they were. We donate to those patients, for everything, and it’s just demand, demand* [...] *nursing suffered a lot of pressure there. There were people who even gave up the service, ended up resigning* [...]. (T31-COVID-19 Inp./public)

[...] *now we are seeing many certificates due to exhaustion. We are no longer seeing absences because of COVID-19, but because people can no longer bear this pressure and work overload. They can’t stand to continue in this environment* [...]. (T34-COVID-19 ICU/public)

Finally, some nursing professionals highlighted that their experiences of suffering were enhanced by the lack of professional recognition and appreciation. Despite the media visibility that occurred for a period, they were frustrated with the precariousness of working conditions and, sometimes, by the neglect of institutions:

[...] *neglect with those who were there* [front line]. *For me, the biggest psychological impact was that. I don’t see anyone recognizing the professionals who worked there* [COVID-19 unit], *especially nurses.* [...] *not everyone lives on applause.* [...] *the salary does not change, the recognition is zero.* [...] *I worked at H1N1 too, it was the same thing, nothing changed* [...]. (T29-COVID-19 ICU/philanthropic)

[...] [co-worker] *showed symptoms, I was with her. It was at the Emergency Room, the X-ray showed a spot, the doctor told her to go back to work; if it got worse, she would take some measure. I had to make a fuss for her to be released, because she wasn’t well* [...] *even so she stayed in the unit for a while to help me, because she was sorry to leave me alone.* (T26-COVID-19 Inp./philanthropic)

*I joined* [the hospital] *because they needed more staff. Today I see how bad it is. How much good co-workers are lost because they don’t adapt. Who is subject to this?* [...]. (T2-COVID-19 Emerg./philanthropic)

Data indicate that there were experiences of suffering. These experiences are mainly related to patients’ morbidity and mortality, but also to individual aspects of workers and those linked to the process, work organization and professional development.

### Impacts of work on the front line: between disenchantment and formation of meanings

The previous analytical categories revealed that nursing work in COVID-19 hospital units was complex, as it involved concomitant experiences of pleasure and suffering. In some moments, the experience of composing the front line was edifying, it attributed meanings to the professional identity, rescuing the formation of meanings for work in nursing and, therefore, allowing a subjective movement of falling in love (again) with the profession:

[...] *I always wanted to study History and started this year. Because I wanted to earn more, have 60 days of vacation. When the pandemic started, at the same time that I was afraid, I also knew that I couldn’t run away from the fight* [...] *I stopped college, I don’t know if I’ll go back* [...] *I don’t want to leave nursing anymore, this is where I want to stay. I’m in the right place. This part of helping is very important to me. It’s not just the money that counts, it’s not just the bigger vacation that counts. That part of knowing I’m in the right place. I like History, but being here* [...] *I was born for this. Priceless* [...]. (T12-COVID-19 Emerg./philanthropic)

However, the way in which these experiences impact nursing workers’ mental health, subjectivity and lives was unique. For another respondent, being on the front line was a catalyst for disenchantment and the desire to no longer practice the profession:

[...] *I’m at the beginning of my career as a* [nursing] *technician, I was really upset, because I created that bond with the patient* [...] *on the first shift I was bagging the guy, he had died. It’s been very difficult psychologically* [...] *it’s stressful, I’m really tired. I don’t know if I will continue in the profession.* [...] *I was going to do nursing now, college. But I think I’ll opt for physical education.* [...] *I’m saturated. I like to help, I really like the contact with the patient, but mainly having caught this pandemic, I think it* [affected] *the psychological a lot* [...]. (T14-COVID-19 Emerg./philanthropic)

Faced with intense feelings related to work in a major health crisis such as COVID-19, professionals reinforced the impact of these experiences on their mental health and the process of subjective and professional transformation that broke out from the front line, at times, towards suffering, and at others, towards growth:

[...] *there’s no way I can look at you and say that my psychology will be the same. Not going anymore! I will never be the same person again, for everything I see there* [COVID-19 unit], *for everything I live* [...]. (T8-COVID-19 ICU/philanthropic)

[...] *I contaminated myself, I was removed. I felt what they* [patients] *feel. After I contaminated myself, I saw that it is much more difficult.* [...] *afterwards, when I came back, I was crying a lot. I put myself in people’s shoes, because they entered and never left. I saw the separation of families that would never see each other again.* [...] *today I feel more prepared. I have a lot of knowledge* [...] *I know how to guide people and be agile in the service* [...] *the emotional side improved with the knowledge we acquired* [...]. *Today I feel much more prepared. Much more frontline!* (T11 - COVID-19 Emerg./philanthropic)

Finally, it should be noted that some testimonies signaled damage to nursing workers’ mental health, showing signs of occupational illness:

[...] *I’ve been having a lot of insomnia.* [...] *a very great sadness* [...] *they are lives, they are families that lose loved ones and it is sad* [...] *it shakes us* [...]. (T35-COVID-19 Inp./public)

[...] *the team will get sick seeing people dying, seeing people going and you not succeeding. How much O2 you offer is not being enough and you don’t have a respirator. You experience things like this. It’s someone’s love that’s there and you think it could be your love* [...] *we try to channel it* [...] *we try to deal, but I know that, for me, one hour will hit harder* [...]. (T10-COVID-19 Sectors/philanthropic)

In the counterpoint between pleasure and suffering, nursing workers at COVID-19 units are impacted in different ways. For some, the experiences of pleasure culminated in the formation of meanings of work. However, others experienced disenchantment, the desire to abandon the profession and signaled the imminence of psychological damage from frontline work.

## DISCUSSION

The first analytical category reveals that work on the front lines of coping with the COVID-19 pandemic provided nursing workers with a set of experiences of pleasure. The representativeness of public and private hospitals and the different sectors in the statements indicates that these experiences are common to the institutions that participated in this study. Firstly, professional gratification for composing the front line was listed, a result similar to that found in a study with emergency nursing workers, whose experiences of pleasure were linked to professional satisfaction and identification with work content^(12)^.

Identification with work content is evident when workers recognize themselves in the role of those who carry out care, considered the central axis of the profession. Similar results were found in other qualitative studies carried out with hospital nursing teams, in which the affinity with the nature of work was referred to as an important experience of pleasure^(13-14)^. The PDW rescues the concept of symbolic resonance, understood as the compatibility between the symbolic representations of individuals and the reality of work, which occurs when work content incorporates a meaning for individuals and their life history^(6-7)^.

This refers to the testimonies of nursing workers, which point to the positive results of their work on the front line (patients’ recovery). Qualitative studies carried out with hospital nursing workers also indicated that the recovery of patients operated as an experience of pleasure^(12-14)^.

These experiences strengthen the feeling of pride for being an important part of the front line of the fight against COVID-19. In PDW, it is considered that when workers contribute with their work space, their return is symbolic retribution, a process in which the construction of personal/professional identity and self-realization through work are strengthened^(6)^.

Added to this, the participants signaled the moments when they received recognition from patients, linking them to experiences of pleasure, which converges with the results of other qualitative research conducted with hospital nursing teams^(12-14)^. For PDW, the dynamics of recognition is the central category for the conquest of identity in the social field and in the construction of the meaning of work. Through recognition, the contributions that individuals make to their collective are validated^(6)^.

The moments of coexistence shared between the team, with the establishment of collaboration, integration and affection relationships, were also linked to experiences of pleasure, meeting the results of other studies that investigated pleasure and suffering in nursing^(12-13)^. For PDW, cooperation ensures people’s willingness to work together and collectively overcome work contradictions. Through cooperation, the collective enhances the efficiency of its activities and protects itself against suffering^(5)^.

The first analytical category concludes with statements in which participants recognize acting on the front line as a catalyst for a process of overcoming their own limits, resilience and pride. It is important to point out that, in the context of working on COVID-19, the relationship between threat and challenge is dynamic and subjective. Situations initially assessed as threatening may come to be seen as challenging as a consequence of a coping process that allows a more positive view. Thus, individuals are able to better mobilize their resources to face these situations^(2)^ and, therefore, are capable of transforming experiences of suffering into pleasure.

In contrast to these results, the second analytical category brings experiences of suffering to the fore. Statements by workers from different sectors were representative. However, in the empirical data, philanthropic scenarios predominated. This may be a result of the prevalence of these institutions in this research (since five were included, while the public ones were in number of two). However, it may also be indicative that the experiences of suffering are more acute in these places.

Initially, the narratives related to the daily suffering and deaths of COVID-19 patients stood out, as well as the feeling of helplessness in the face of their clinical decline. Research has indicated that living with death stands out as an important experience of suffering for nursing in hospitals^(12,14)^, especially when there are bonds between workers and patients^(13)^.

In COVID-19 units, nursing workers suffer emotionally from the death of patients and the suffering of their families^(15)^. Qualitative research with Canadian health professionals showed that, in their perception, COVID-19 increased the suffering of patients in the death and dying process, partly due to the absence of their loved ones and, consequently, the loneliness and anguish experienced at the time of death. The study professionals found it difficult to humanize this moment, which gave them feelings of anguish^(16)^.

For PDW, suffering is established when workers mobilize their intellectual, psycho-affective, learning and adaptation capacities to the maximum, but are not able to transform the product of their work in the way they want. In addition to the physical and psycho-affective demands that individuals face in their daily lives, the experience of dissatisfaction and frustration is powerful mechanism of suffering^(6)^.

The daily frustrations related to the clinical decline and death of patients intersected with participants’ personal experiences, especially in situations where there was contamination and death of co-workers and loved ones. A Brazilian qualitative study carried out with nursing workers showed intersections between work experiences and memories of personal experiences, especially related to family issues. Nursing professionals were not always able to manage the complexity of their emotions when perceiving the suffering of the other, aspects that referred them to their own family^(13)^.

PDW considers that life at work and outside of it is a continuum that is difficult to dissociate^(6)^. In the COVID-19 units, workers simultaneously occupy the place of ordinary citizens who share in the human and family losses caused by the pandemic. Managing these losses and being at the front line at the same time is a challenge, because at the same time that you are a reference in the workspace, workers feel a reference in the family nucleus^(17)^.

In addition to issues related to the morbidity and mortality of COVID-19 patients, experiences of suffering related to the organization of work emerged (with emphasis on dissatisfaction with the team, institution, lack of recognition and devaluation). For PDW, the organization of work is at the center of most experiences of suffering, which are evident when individuals do not find spaces for flexibility and negotiation in this organization. Therefore, in these cases, work ceases to be a synergistic space with workers’ individuality^(6)^.

Interviewees signaled lack of collaboration and aggregation of the team at times. It is known that disruptive interpersonal relationships are a reality in nursing teams and that they imply damage to professionals’ well-being and the performance of their work^(18)^. A qualitative study carried out with pediatric oncopediatrics nursing professionals showed that the noise of communication and the management of interpersonal relationships operated as experiences of suffering^(14)^.

Relationships in the workspace were aggravated by the perception that there was overload and excesses in institutional demands. This feeling emerged at a time when, when composing the front line, workers felt that they did not receive the institutional support they needed. Qualitative Brazilian research with nursing workers at an emergency care service showed that some experiences of suffering were related to the lack of support from hospital management^(12)^, supporting these findings.

Added to this was the lack of institutional recognition and professional devaluation. For PDW, when feelings of professional recognition are not established, workers are unable to transform their suffering into pleasure and do not find a meaning for work^(6)^. A study developed with nursing professionals working in COVID-19 units in the Philippines showed that those who perceived greater organizational and social support reported lower levels of anxiety when compared to the others^(19)^. Therefore, it is perceived that institutional and social support are fundamental for job satisfaction and mental health.

Finally, the last analytical category reveals the unique impacts of the front line on workers’ subjectivity. The comparison between the perception of two participants suggests that, while, for some, the work at the COVID-19 units may have allowed them to fall in love (again) with the profession, through the strengthening of meanings of work, for others, the same experience may have culminated in disenchantment and a desire to abandon the profession.

PDW points to the existence of two types of suffering: creative and pathogenic. Creative suffering works as a catalyst for a process of subjective or collective mobilization towards the transformation of work organization and situations that trigger dissatisfaction. Pathogenic suffering, on the other hand, is established when workers do not find resources in the organization of work to face adversities, evolving into the risk of illness^(6)^.

It is important to take a special look at the desire to no longer practice the profession, triggered by the experience of composing the front line. A study carried out with Brazilian hospital nurses showed an association between emotional exhaustion and the desire to stop practicing nursing^(20)^. Moreover, it is known that COVID-19 has exacerbated problems related to nursing workforce retention in the world^(21)^.

On the other hand, it is also important to shed light on the paradoxical (re)encounter with the meaning of work in a period of austerity and loss, such as that of COVID-19. Do you know that the pandemic has exacerbated the precariousness that already exists in the daily lives of nursing professionals around the world. However, being on the front line promoted the incorporation of meanings related to the recognition of the social, political and economic value of nursing. This strengthened, in the subjectivity of many people, the reason for being in this profession^(22)^. Therefore, despite the hard experiences of suffering, those of pleasure could allow work to make (or make) sense again.

Finally, the deleterious impacts of the pandemic on some participants’ mental health are highlighted. A Brazilian study showed the prevalence of anxiety (48.9%) and depression (25%) among nursing workers at a COVID-19 unit^(2)^, which signals the importance of discussing the risks of mental illness among professionals who are on the front line.

COVID-19 highlighted the consequences of the precariousness of the health sector, increasing workers’ psychological distress and negatively impacting their physical and mental health. However, it is hoped that these workers remain physically and mentally healthy, as they are an important part of fighting the pandemic^(9,15,23)^. In addition, when returning to their sectors of origin in the transition to the post-pandemic period, they must find conditions to reframe these experiences of suffering so that mental illness is not a legacy of the front line.

To achieve this, it is expected that what is experienced by nursing workers who work in COVID-19 hospital units has visibility in academia, science, society and in the health care network management. It is hoped that visibility will be converted into appreciation of the category, improvement of working conditions, enhancement of professional autonomy and creation of spaces for the promotion of mental health at work, including in the post-pandemic period.

### Study limitations

This study presented as a limitation the temporality in which it was carried out. Over the course of a year of data production, interviews were conducted at different health stages of the pandemic. With the abrupt changes in the capacity rates of the units and morbidity and mortality rates, the interviews may have reflected aspects related to the moment in which they were produced.

### Contributions to nursing, health, or public policies

The results of this study provide information about the elements that enhance and weaken nursing workers’ mental health at COVID-19 hospital units. By including the perspective of workers from different regions, from public and private medium and large hospitals, and from different sectors, the representativeness of the findings was enriched. It is expected that these results will be used as subsidies for structuring actions to promote mental health and participatory management of the hospital nursing work process.

## FINAL CONSIDERATIONS

The study allowed analyzing the experiences of pleasure and suffering of nursing workers in COVID-19 hospital units. Pleasure experiences were related to personal gratification, identification with work content, positive results obtained in daily care, recognition, coexistence/integration with the team, resilience and personal overcoming. Suffering, on the other hand, was linked to daily life of losses and deaths, helplessness in the face of clinical decline of patients, loss of co-workers and loved ones due to COVID-19, lack of team collaboration, institutional demands, lack of appreciation and recognition.

The dynamics between the experiences of pleasure and suffering mobilized workers, sometimes for a reunion with the meanings of work, sometimes for disenchantment and the desire to leave the profession. It is possible to conclude that, in the dynamics between pleasure and suffering in nursing work in COVID-19 hospital units, elements point to the risk of psychological illness.
